# Lymphocyte count polygenicity underlies dimethyl fumarate–associated lymphopenia in multiple sclerosis

**DOI:** 10.1177/13524585261471335

**Published:** 2026-08-17

**Authors:** Kaarina Kowalec, Ali Manouchehrinia, Elaine Kingwell, Robert Carruthers, Ruth Ann Marrie, Charity Evans, Dalia Rotstein, Annie J Kuan, Wenzhuo Luo, Daniel Luo, Brianne Desrochers, Vilija G Jokubaitis, Jeannette Lechner-Scott, Vicki E Maltby, Kira Groen, Galen EB Wright, Britt I Drögemöller, Sasha Bernatsky, Michael C Levin, Katherine Knox, Abdullah Al Maruf, Michael Zhong, Lars Alfredsson, Jan Hillert, Ingrid Kockum, Tomas Olsson, Pernilla Stridh, Klementy Shchetynsky, Kristy Dever, Julie MacIsaac, Michael S Kobor, Helen Tremlett

**Affiliations:** College of Pharmacy, University of Manitoba, Winnipeg, MB, Canada; Department of Biochemistry & Medical Genetics, Max Rady College of Medicine, University of Manitoba, Winnipeg, MB, Canada; Department of Medical Epidemiology & Biostatistics, Karolinska Institutet, Stockholm, Sweden; College of Pharmacy, University of Manitoba, Winnipeg, MB, Canada; Karolinska Neuroimmunology and Multiple Sclerosis Center, Department of Clinical Neuroscience, Karolinska Institutet, Stockholm, Sweden; Department of Primary Care and Population Health, Institute of Epidemiology and Health Care, University College London, London, UK; Department of Medicine, University of British Columbia, Vancouver, BC, Canada; Department of Medicine and Community Health and Epidemiology, Faculty of Medicine, Dalhousie University, Halifax, NS, Canada; Nova Scotia Health, Halifax, NS, Canada; Department of Internal Medicine, Max Rady College of Medicine, Rady Faculty of Health Sciences, University of Manitoba, Winnipeg, MB, Canada; College of Pharmacy and Nutrition, University of Saskatchewan, Saskatoon, SK, Canada; Department of Medicine, Division of Neurology, University of Toronto, Toronto, ON, Canada; Faculty of Medicine (Neurology), University of British Columbia, Vancouver, BC, Canada; College of Pharmacy, University of Manitoba, Winnipeg, MB, Canada; College of Pharmacy, University of Manitoba, Winnipeg, MB, Canada; College of Pharmacy, University of Manitoba, Winnipeg, MB, Canada; Department of Neuroscience, School of Translational Medicine, Monash University, Melbourne, VIC, Australia; Department of Neurology, Alfred Health, Melbourne, VIC, Australia; Department of Neurology, John Hunter Hospital, Newcastle, NSW, Australia; School of Medicine and Public Health, University of Newcastle, Newcastle, NSW, Australia; Immune Health Research Program, Hunter Medical Research Institute, Newcastle, NSW, Australia; Department of Neurology, John Hunter Hospital, Newcastle, NSW, Australia; School of Medicine and Public Health, University of Newcastle, Newcastle, NSW, Australia; Immune Health Research Program, Hunter Medical Research Institute, Newcastle, NSW, Australia; Immune Health Research Program, Hunter Medical Research Institute, Newcastle, NSW, Australia; Department of Biochemistry & Medical Genetics, Max Rady College of Medicine, University of Manitoba, Winnipeg, MB, Canada; Department of Pharmacology and Therapeutics, Rady Faculty of Health Sciences, University of Manitoba, Winnipeg, MB, Canada; PrairieNeuro Research Centre, Kleysen Institute for Advanced Medicine, Health Sciences Centre and Rady Faculty of Health Sciences, University of Manitoba, Winnipeg, MB, Canada; Children’s Hospital Research Institute of Manitoba, Winnipeg, MB, Canada; Department of Biochemistry & Medical Genetics, Max Rady College of Medicine, University of Manitoba, Winnipeg, MB, Canada; Children’s Hospital Research Institute of Manitoba, Winnipeg, MB, Canada; CancerCare Manitoba Research Institute, Winnipeg, MB, Canada; Centre on Aging, Winnipeg, MB, Canada; Department of Medicine, Divisions of Clinical Epidemiology and Rheumatology, Research Institute of the McGill University Health Centre, Montreal, QC, Canada; Office of the Saskatchewan Multiple Sclerosis Research Chair, Department of Medicine, Division of Neurology, College of Medicine, University of Saskatchewan, Saskatchewan, Saskatoon, SK, Canada; Department of Anatomy, Physiology and Pharmacology, College of Medicine, University of Saskatchewan, Saskatoon, SK, Canada; Department of Physical Medicine and Rehabilitation, College of Medicine, University of Saskatchewan, Saskatoon, SK, Canada; College of Pharmacy, University of Manitoba, Winnipeg, MB, Canada; Children’s Hospital Research Institute of Manitoba, Winnipeg, MB, Canada; Department of Psychiatry, Max Rady College of Medicine, University of Manitoba, Winnipeg, MB, Canada; Department of Neuroscience, School of Translational Medicine, Monash University, Melbourne, VIC, Australia; Department of Neurology, Alfred Health, Melbourne, VIC, Australia; Karolinska Neuroimmunology and Multiple Sclerosis Center, Department of Clinical Neuroscience, Karolinska Institutet, Stockholm, Sweden; Institute of Environmental Medicine, Karolinska Institutet, Stockholm, Sweden; Karolinska Neuroimmunology and Multiple Sclerosis Center, Department of Clinical Neuroscience, Karolinska Institutet, Stockholm, Sweden; Karolinska Neuroimmunology and Multiple Sclerosis Center, Department of Clinical Neuroscience, Karolinska Institutet, Stockholm, Sweden; Karolinska Neuroimmunology and Multiple Sclerosis Center, Department of Clinical Neuroscience, Karolinska Institutet, Stockholm, Sweden; Center for Molecular Medicine, Karolinska University Hospital, Stockholm, Sweden; Karolinska Neuroimmunology and Multiple Sclerosis Center, Department of Clinical Neuroscience, Karolinska Institutet, Stockholm, Sweden; Center for Molecular Medicine, Karolinska University Hospital, Stockholm, Sweden; Karolinska Neuroimmunology and Multiple Sclerosis Center, Department of Clinical Neuroscience, Karolinska Institutet, Stockholm, Sweden; Center for Molecular Medicine, Karolinska University Hospital, Stockholm, Sweden; Edwin SH Leong Centre for Healthy Aging, Faculty of Medicine, University of British Columbia, Vancouver, BC, Canada; Centre for Molecular Medicine and Therapeutics and Department of Medical Genetics, University of British Columbia, Vancouver, BC, Canada; British Columbia Children’s Hospital Research Institute, Vancouver, BC, Canada; Edwin SH Leong Centre for Healthy Aging, Faculty of Medicine, University of British Columbia, Vancouver, BC, Canada; Centre for Molecular Medicine and Therapeutics and Department of Medical Genetics, University of British Columbia, Vancouver, BC, Canada; British Columbia Children’s Hospital Research Institute, Vancouver, BC, Canada; Edwin SH Leong Centre for Healthy Aging, Faculty of Medicine, University of British Columbia, Vancouver, BC, Canada; Centre for Molecular Medicine and Therapeutics and Department of Medical Genetics, University of British Columbia, Vancouver, BC, Canada; British Columbia Children’s Hospital Research Institute, Vancouver, BC, Canada; Faculty of Medicine (Neurology), University of British Columbia, Vancouver, BC, Canada; The Djavad Mowafaghian Centre for Brain Health, Vancouver, BC, Canada

**Keywords:** Multiple sclerosis, dimethyl fumarate, lymphopenia, polygenic score, pharmacogenomics, adverse drug reaction

## Abstract

**Purpose:**

One in 20 people using dimethyl fumarate (DMF), a commonly used disease-modifying therapy for multiple sclerosis (MS), develops severe lymphopenia. We aimed to identify genetic variation associated with DMF-related lymphopenia.

**Methods:**

Using a case–control design, we included cases that had either grade 2 (absolute lymphocyte count <0.8 × 10^9^/L) or 3 (<0.5 × 10^9^/L) lymphopenia during DMF. Controls had normal lymphocyte counts and ⩾1 year of DMF exposure. We generated a polygenic score for lymphocyte counts and evaluated its ability to predict DMF-associated lymphopenia in two cohorts, with adjustment for covariates. We also conducted a genome-wide association study (GWAS) and gene-based analyses.

**Findings:**

The discovery cohort comprised 238 PwMS of European genetic ancestry from Canada/Australia (*n* = 72 grade 2 cases, 34 grade 3 cases), and the replication cohort comprised 156 PwMS from Sweden (*n* = 34 grade 2, 14 grade 3). A higher polygenic score for lymphocyte counts predicted reduced risk of grade 2 lymphopenia (hazard ratio = 0.43, 95% confidence interval: 0.25–0.73). The GWAS identified one locus associated with the minimum lymphocyte count during DMF (rs17087205, beta = −0.63, SE = 0.11, *p* = 3.89e–8) but did not replicate (beta = −0.04, *p* = 0.47).

**Conclusion:**

Common polygenic variation of lymphocyte counts was significantly associated with moderate lymphopenia during DMF treatment.

## Introduction

The commonly used first-line oral disease-modifying therapy for multiple sclerosis (MS), dimethyl fumarate (DMF), has been associated with progressive multifocal leukoencephalopathy (PML) and other opportunistic infections.^1^ PML represents a serious, potentially fatal adverse event that has restricted the use of some MS therapies.^2^ Currently, there are few clinically useful predictors of opportunistic infections in persons with MS taking DMF.^3^

One risk factor for PML in persons taking DMF is lymphopenia, a reduction in lymphocyte counts below the normal range.^4^ Approximately 5%–6% of DMF-randomized participants with MS developed very low counts (<0.5–0.2 × 10^9^/L) in trials, compared to <1% on placebo.^5^ Regular lymphocyte screening remains the main strategy to identify reductions,^6^ yet even combined with demographic and clinical factors, it does not reliably predict lymphopenia sufficiently for primary prevention.

Genetic variation can stratify individuals with respect to adverse drug reaction risk, and replicated genetic biomarkers have been identified for other drug reactions, including interferon-beta–induced liver injury in MS.^7^ Polygenic scores capture the weighted sum of all the genetic risk alleles,^8^ with their use in pharmacogenetics increasing.^9^

A 2019 study authored by employees of the drug manufacturers and marketers of DMF investigated the genetic determinants of DMF-induced lymphopenia from people with MS enrolled in trials.^10^ The authors concluded absolute lymphocyte counts remained the most effective way of identifying those at risk of severe DMF-associated lymphopenia.^10^ Differences between trial participants and real-world patients^11^ combined with the prior focus on single variants rather than polygenicity motivate this study.

We aimed to determine whether the genetic architecture underlying lymphocyte counts in the general population is associated with DMF-associated lymphopenia in MS and performed a genome-wide association study (GWAS) to identify associated variants. Identifying those at genetic risk of developing lymphopenia before DMF could enable earlier surveillance or pretreatment stratification, with potential relevance beyond MS to other therapies associated with lymphopenia.

## Materials and methods

### Study design and populations

We used a case–control study design with two groups: discovery and replication. Participants from Canada or Australia formed the discovery cohort, while participants from Sweden formed the replication cohort (details in the Supplemental Material). Our study protocol was published.^12^ All participants provided informed consent. The study was approved by the respective site Health Research Ethics Boards and Health Authorities.

### Participant criteria and case/control definitions

All participants were ⩾18 years, diagnosed with relapsing-onset MS (based on the contemporary criteria at enrolment) and had documented DMF exposure, with a baseline, pre-DMF absolute lymphocyte count test result within the normal range.

Cases were defined as persons with MS who developed lymphopenia at any time during DMF treatment. Prior use of other MS disease-modifying therapies was not an exclusion criterion. Grade 2 lymphopenia was defined in an individual if, during DMF exposure, they had an absolute lymphocyte count of <800/mm^3^ or <0.8 × 10^9^/L on ⩾1 laboratory blood test and grade 3 lymphopenia as <500/mm^3^ or <0.5 × 10^9^/L on ⩾1 laboratory blood test during DMF exposure. Prolonged lymphopenia is also clinically relevant^1^ and was defined as two or more test results at least 6 months apart indicating grade 2 (<800/mm^3^ or <0.8 × 10^9^/L) or grade 3 (<500/mm^3^ or <0.5 × 10^9^/L) lymphopenia. All cases meeting the grade 3 lymphopenia definition would, by definition, also be included as cases of grade 2 lymphopenia. Our primary comparison was grade 2 lymphopenia versus DMF-tolerant controls. Second, we compared grade 3 lymphopenia or prolonged lymphopenia to DMF-tolerant controls. DMF-tolerant controls were defined as individuals with MS who had at least 1 year of DMF exposure,^13^ and all available on-treatment lymphocyte counts within the normal laboratory reference range, with a minimum of one on-treatment count required. Counts were not restricted to a single timepoint; all post-baseline measurements recorded during DMF exposure were used to confirm sustained tolerance across the observation period. DMF-tolerant controls were preferred over population controls (with unknown drug exposure) as they provide the greatest statistical power while minimizing confounding in a case–control GWAS of adverse drug reactions.^14^ We also examined the minimum lymphocyte count during DMF treatment as a continuous outcome. For this analysis, we used the log-transformed minimum lymphocyte count as the phenotype in the PLINK2 linear regression analyses.

### Exposures

Details of genotype processing are available (Supplemental Material). We investigated individual genetic variants in the GWAS, conducted gene-based and pathway enrichment analyses (Supplemental Material) and then also computed polygenic scores. Polygenic scores are the weighted sum of the number of known genetic variants associated with a given trait (e.g. lymphocyte counts) within each person. We generated the polygenic scores for lymphocyte counts using summary-level existing GWAS data from the Pan-UK Biobank project, which included 407,277 European ancestry individuals and their measured lymphocyte count^15^ (Pan-UK Biobank phenocode: 30120). We computed the scores in our study participants (Canada/Australia, Sweden) using a Bayesian multiple regression approach (SBayesR)^16^ and PLINK1.9 and the existing GWAS data. The resulting polygenic score is a continuous, individual-level measure that captures cumulative genetic liability for lymphocyte counts and was standardized to a mean = 0 and a standard deviation = 1.

### Statistical analyses

Analyses were conducted separately in the discovery and replication cohorts. We evaluated whether a polygenic score for lymphocyte count predicted the development of lymphopenia during DMF exposure in MS. For each participant, serial absolute lymphocyte counts were compiled and aligned by months since DMF initiation. To capture the timing of lymphopenia onset, interval-censored survival data were constructed for each lymphopenia grade, classifying observations as interval- or right-censored depending on whether the event occurred between two visits or was not observed during follow-up. Semiparametric Cox proportional hazards models for interval-censored data were then fitted to estimate the association between the polygenic score, analysed both as a standardized continuous variable and as a categorical variable contrasting the top 20% of the polygenic score distribution with the lower 80% of the distribution, and time to first occurrence of the specified lymphopenia grade. The grade 2 outcome was defined as the first occurrence of absolute lymphocyte count <0.8 × 10^9^/L during DMF exposure and, therefore, included participants who also subsequently met the grade 3 threshold (<0.5 × 10^9^/L); grade 3 lymphopenia was analysed separately as the more severe threshold. We computed two models based on adjustment for different confounders: (1) age at DMF initiation, sex and the first five genetic ancestry principal components and (2) the same confounders as model 1 plus baseline lymphocyte count. We report the hazard ratios (HRs) and 95% confidence intervals (CIs); CIs were estimated using 1000 bootstrap samples. Participants who did not reach the grade-specific threshold were treated as right-censored at their last observation. In the Swedish cohort, we only performed grade 2 analysis due to the small number of grade 3 events and unstable model outputs. The proportional hazards assumption was assessed approximately by fitting Cox models to single-time representations of the interval-censored data and evaluating Schoenfeld residual-based tests. For the Cox models, all *p* values were two-sided, and *p* values <0.05 were considered statistically significant.

## Results

In the discovery cohort, after quality control and following the removal of genetic ancestry outliers (Supplemental Figure S1), 238 individuals of European genetic ancestry remained. The replication cohort (Sweden) included 156 individuals of European genetic ancestry after applying the inclusion and exclusion criteria and removing the genetic ancestry outliers. Participants in the discovery and replication cohorts were similar in terms of their characteristics (Table 1).

**Table 1.** Study population characteristics of the European genetic ancestry participants with multiple sclerosis (MS) exposed to dimethyl fumarate (DMF) treatment.

	Discovery cohort: Canada and Australia	Replication cohort: Sweden
*N*	238	156
DMF-tolerant controls, *n*	116	122
Age at MS onset, median (IQR)	31 (25, 37)	32 (26, 39)
Age at DMF start, median (IQR)	43 (34, 50)	40 (33, 49)
Female sex (*n*, %)	181 (76%)	111 (71%)
Length of DMF treatment, median days (IQR)	539 (273–970)	450 (360–630)
Lowest absolute lymphopenia counts during DMF treatment, median (IQR)	0.9 (0.6–1.3)	0.9 (0.7–1.3)
Lymphopenia grade during DMF treatment (*n*, %)		
Grade 2 (<0.8 × 10^9^/L)	72 (30%)	34 (22%)
Grade 3 (<0.5 × 10^9^/L)	34 (14%)	14 (9%)
Prolonged lymphopenia during DMF treatment (*n*, %)^a^		
Grade 2 (<0.8 × 10^9^/L)	45 (19%)	13 (8%)
Grade 3 (<0.5 × 10^9^/L)	11 (4.6%)	2 (1%)

a Individuals with ⩾2 test results at least 6 months apart indicating lymphopenia (absolute lymphocyte counts <500/mm^3^/<0.5 × 10^9^/L for grade 3 and <800/mm^3^/<0.8 × 10^9^/L for grade 2).

The mean polygenic scores by case/control status are presented in Figure 1. In the discovery cohort, a higher polygenic score was significantly associated with a lower risk of developing lymphopenia following DMF initiation (Figure 2). When modelled as a continuous standardized variable, the polygenic score (one standard deviation increase) was associated with a 57% reduction in grade 2 lymphopenia (adjusted HR = 0.43, 95% CI: 0.25–0.73, *p* = 0.002, Figure 2). The categorical model comparing the top 20% versus the lower 80% of polygenic score values yielded similar estimates but with broader CIs (grade 2 adjusted HR = 0.58, 95% CI: 0.33–1.02, Figure 2). No statistically significant association was observed for grade 3 lymphopenia, likely reflecting the fewer events. In the independent Swedish replication cohort, results were directionally consistent with the discovery (Figure 2).

**Figure 1.**
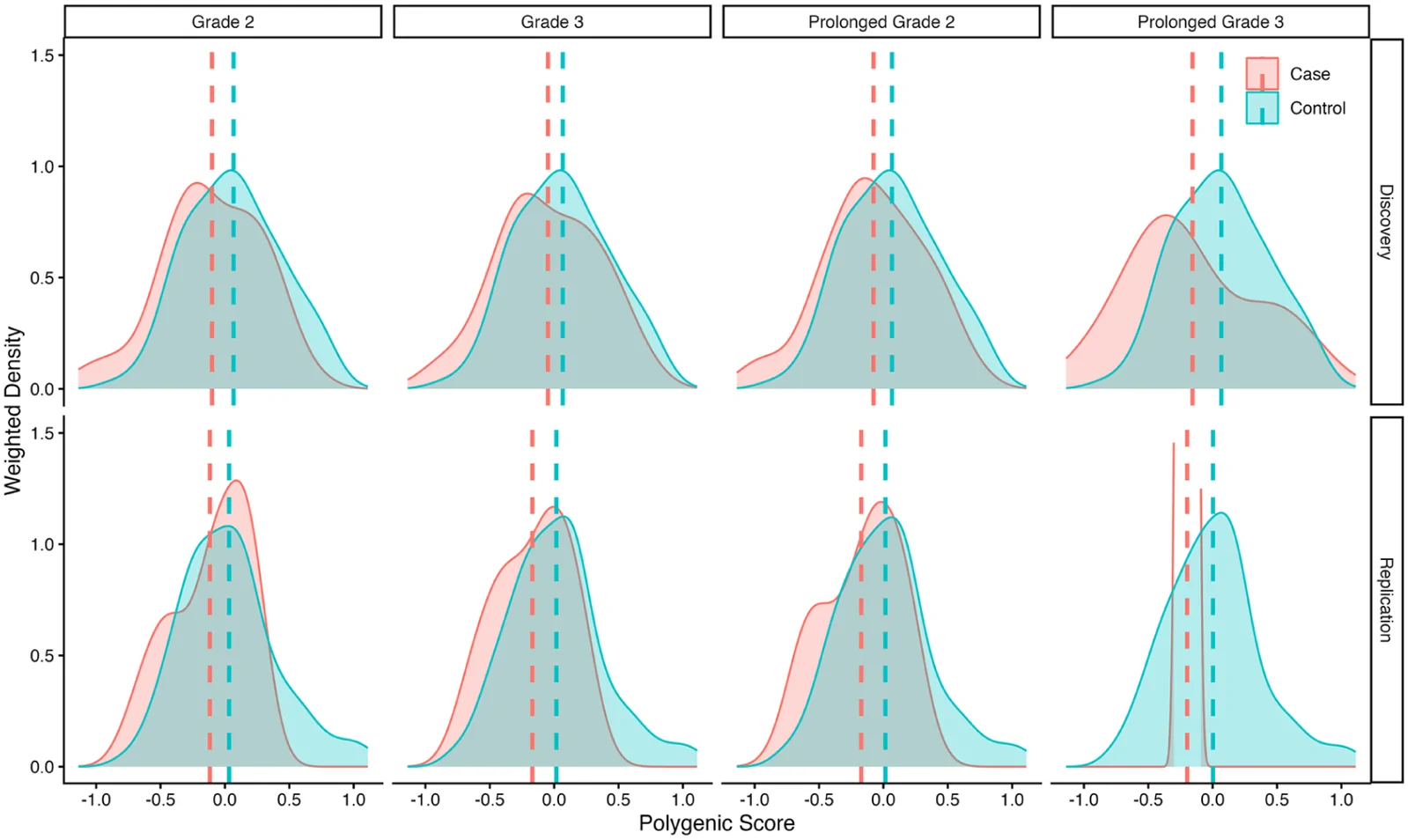
Weighted density plots of the lymphocyte count polygenic score by comparison in people with multiple sclerosis exposed to dimethyl fumarate treatment in the discovery and replication cohorts. Each plot contains a dotted line representing the mean lymphocyte count polygenic score by case or control. Absolute lymphocyte count by grade: grade 2: <800/mm^3^/<0.8 × 10^9^/L and grade 3: <500/mm^3^/<0.5 × 10^9^/L. Prolonged grade 2 represents individuals with ⩾2 test results at least 6 months apart indicating that level of lymphopenia. The discovery cohort includes participants from Canada or Australia, and the replication cohort is from Sweden.

**Figure 2.**
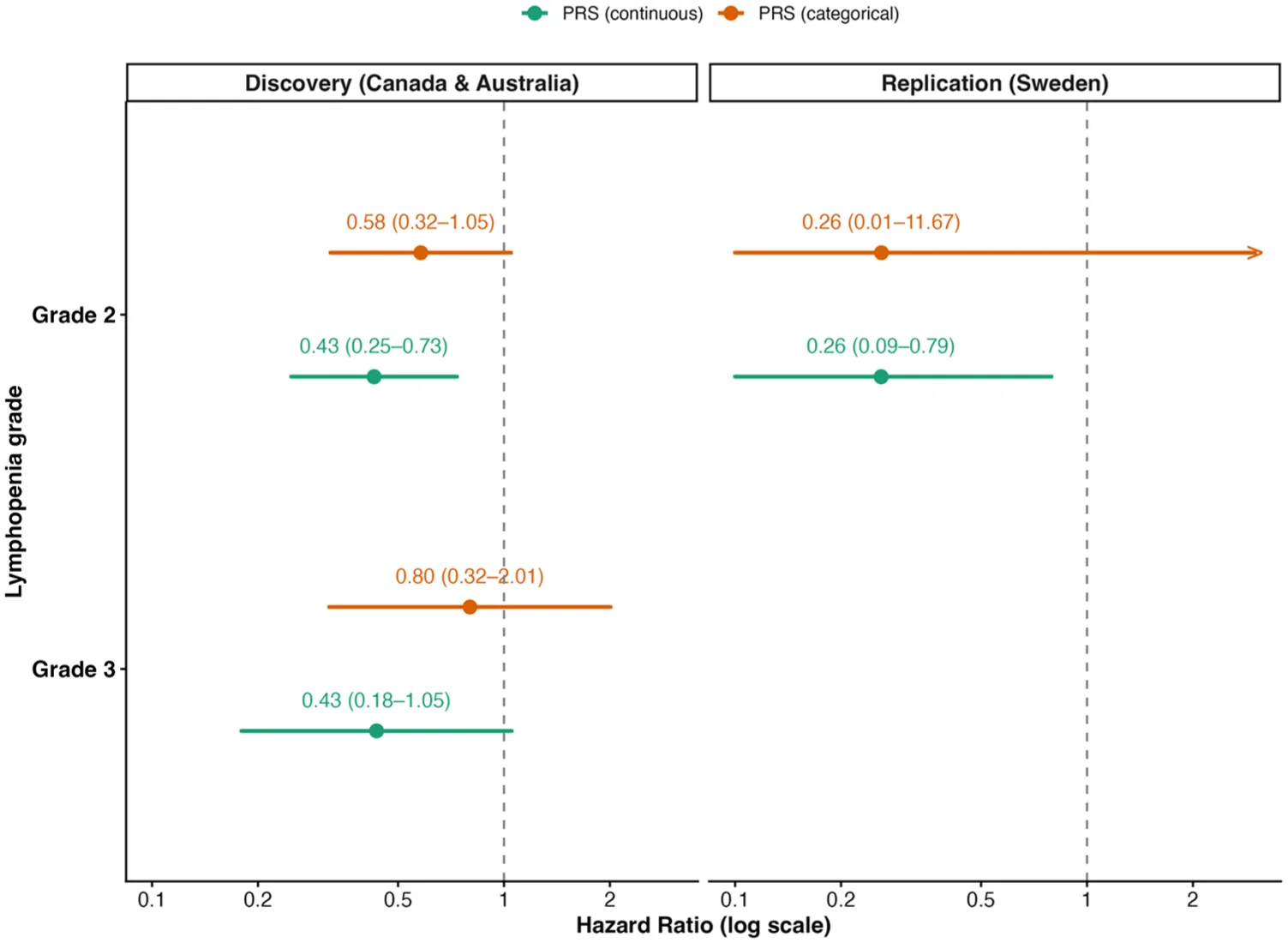
Cox regression analyses investigating the association between lymphocyte count polygenic score and the hazard of developing different grades of lymphopenia during dimethyl fumarate treatment in multiple sclerosis, by cohort. The model did not converge for grade 3 lymphopenia in the Swedish cohort due to the small number of events (*n* = 2 cases with grade 3 lymphopenia).

To assess whether the genetic association persisted and offered predictive value beyond individual differences in pre-DMF treatment lymphocyte levels, we incorporated baseline lymphocyte count into the modelling (Supplemental Table S1). In the discovery cohort, the baseline lymphocyte count was inversely associated with the risk of lymphopenia, with lower pre-DMF lymphocyte counts predicting a higher risk of lymphopenia (grade 2: adjusted HR = 0.54, 95% CI: 0.28–1.03, *p* = 0.06; grade 3: adjusted HR = 0.49, 95% CI: 0.27–0.90, *p* = 0.02), the protective effect of the higher lymphocyte polygenic score remained evident and statistically significant for grade 2 lymphopenia (adjusted HR = 0.48, 95% CI: 0.27–0.87, *p* = 0.016) and directionally consistent but not significant for grade 3 lymphopenia (adjusted HR = 0.58, 95% CI: 0.24–1.39, *p* = 0.22). In the Swedish replication cohort, results were again in a similar direction to that of the discovery sample, although the model for grade 3 lymphopenia did not converge due to a very small number of events (Supplemental Table S1).

We identified one genome-wide significant variant on chromosome 9 in association with the lowest absolute lymphocyte count during DMF treatment in the discovery (Table 2, Figure 3, Supplemental Figure S2, Supplemental Table S2). This resulted in a single LD-independent significant locus (*r*^2^ < 0.1 within 3 Mb) and one region after merging loci within 50 kb with 99 variants (Supplemental Figure S3). We assessed the effect of this variant with the lowest lymphocyte count during DMF treatment in the replication cohort, and although it was in the same direction, it was not significant (beta = −0.04, *p* = 0.47, Supplemental Figure S5). We did not identify any genome-wide significant variants in association with grade 2 or 3 or prolonged grade 2 or 3 lymphopenia (Supplemental Figures S4–S6, Supplemental Tables S3–S7).

**Table 2.** Genetic association results for the lowest lymphocyte count during dimethyl fumarate treatment in people with multiple sclerosis.

Locus information				Discovery cohort: Canada/Australia (*n* = 238)			Replication cohort: Sweden (*n* = 156)		
ID	Chromosome: Position	Allele 1/Allele 2	Start, end	Allele 1 frequency	Beta (SE)	*p*	Allele 1 frequency	Beta (SE)	*p*
rs17087205	9:84,417,739	G/A	84,375,227, 84,479,152	0.055	−0.63 (0.11)	3.89e–8	0.088	−0.05 (0.09)	0.62

All coordinates are in the genome build GRCh38/hg38. For reference, the rs17087205 genomic position in GRCh37/hg19 coordinates is as follows: 9:87,032,654. SE: standard error.

**Figure 3.**
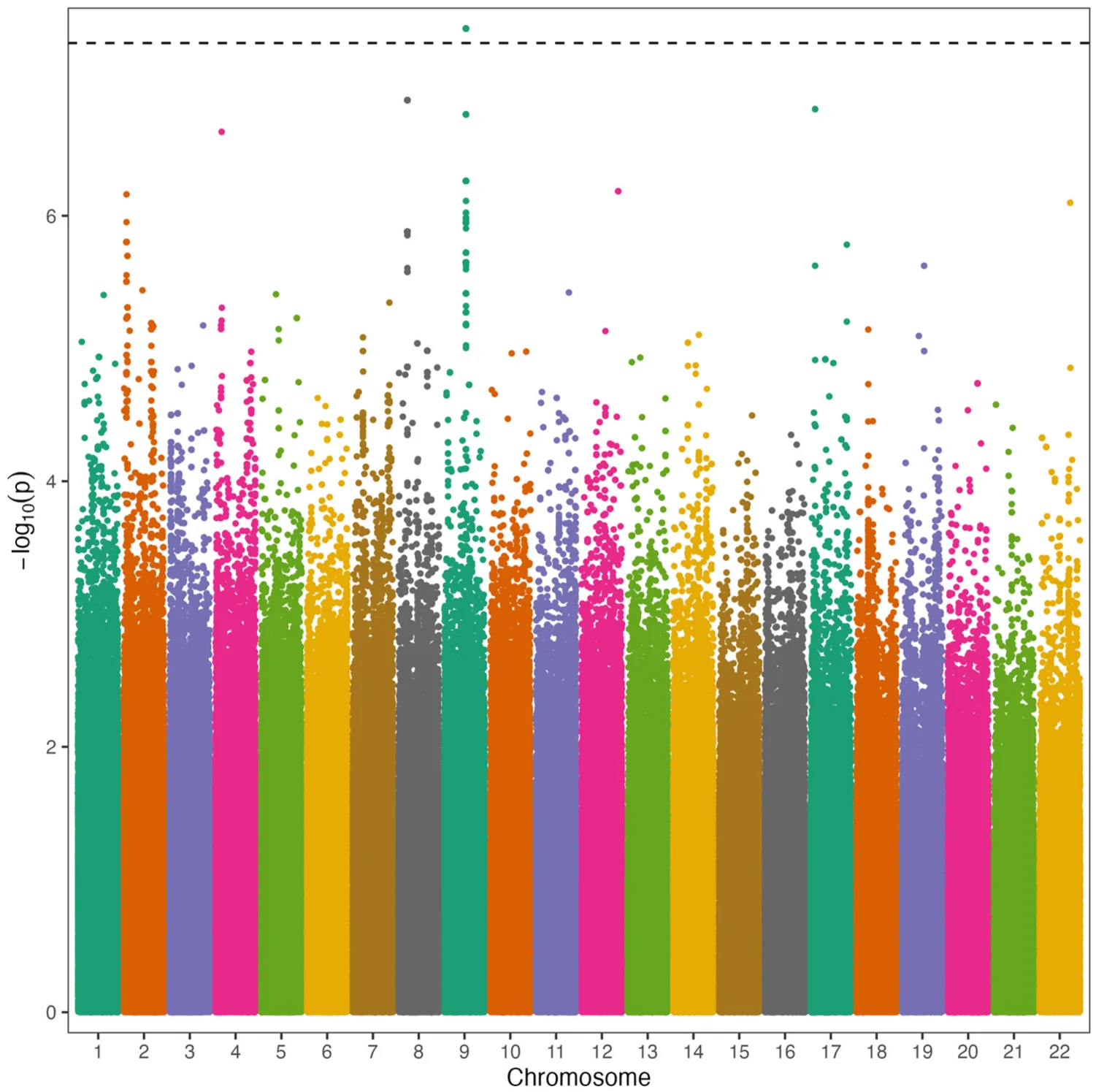
Manhattan plot for the discovery cohort depicting the genome-wide association results for the lowest lymphocyte count measured in 238 people with multiple sclerosis treated with dimethyl fumarate. Black-dashed line (genome-wide significance): *p* < 5 × 10^−8^.

We assessed the effect of a genetic variant previously associated with DMF-associated lymphopenia,^17^ albeit just below the standard genome-wide significance threshold in the previous study, with our study comparisons (grade 2, grade 3, prolonged grade 2 and prolonged grade 3) in the discovery cohort. The effect of this variant was in the same direction, but it was again not significant (Supplemental Table S7).

### Gene-based and pathway enrichment analyses

Across 18,051 tested genes, no gene reached Bonferroni-corrected or FDR significance (minimum gene-level False Discovery Rate (FDR) was approximately 0.34); top-ranked genes were *PRKAG3, LPL, SCN9A, LILRB2* and *SLC28A3* (Supplemental Table S8). Competitive pathway enrichment analyses using MSigDB Hallmark, Reactome and Gene Ontology gene sets identified no pathway surviving global FDR correction across all collections (data not shown).

## Discussion

DMF, an effective disease-modifying therapy used first-line to treat MS in many world regions, is associated with lymphopenia, making it highly relevant to identify how to prevent severe lymphopenia.^1,18^ Using polygenic scores, we found genetic liability for lower lymphocyte count was significantly associated with multiple indicators of lymphopenia in DMF-treated people with MS in the discovery and replication cohorts. This effect was apparent even with adjustment for known risk factors for DMF-associated lymphopenia, including baseline lymphocyte counts. This suggests genetic predisposition influences lymphopenia risk even after accounting for pretreatment lymphocyte levels. In addition, we discovered a common genetic variant significantly associated with the lowest lymphocyte count during DMF treatment, although this did not replicate in the cohort from Sweden.

Traits such as adverse drug reactions are polygenic, and the cumulative genetic inheritance per individual can be captured using a polygenic score.^8,19^ Polygenic scores utilize training data; in our case, GWAS data generated from the UK Biobank, including genotypes and lymphocyte counts on >400,000 individuals,^15^ to calculate an individual’s cumulative inheritance for lymphocyte counts. Studies on weight gain during treatment with antidepressants or antipsychotics have found that those with higher body mass index (BMI) polygenic scores were at greater risk for weight gain, irrespective of the antidepressant or antipsychotic used.^20,21^ Our results converge with these findings as it underscores a consistent picture of genetic architecture: polygenicity for a biological trait, such as lymphocyte levels or BMI, can sensitize an individual to drug-related disruption of that same trait. This effect was observed in our study and in studies of unrelated drug classes such as antidepressants or antipsychotics and highlights a potentially intrinsic, genetically related trait vulnerability to treatment-induced adverse reactions or effects.

We found an increased genetic liability in people with MS for moderate lymphopenia (grade 2) associated with DMF treatment. Those with a genetic liability for lower lymphocyte counts were more likely to develop moderate lymphopenia during DMF treatment, even when baseline counts fell within the laboratory reference range. The association observed for grade 2 lymphopenia should be interpreted as reflecting susceptibility to DMF-related lymphocyte decline more broadly. Grade 2 represents a clinically actionable window: it was common enough to permit well-powered analysis and occurs early enough that intervention remains possible, before the severe immunosuppression and opportunistic infection risk associated with grade 3. The absence of a statistically significant association for grade 3 alone likely reflects the smaller number of severe events and consequent loss of precision. Because this polygenic score is derived from germline genetic data, it could in principle be applied at the time of DMF initiation to identify patients warranting closer lymphocyte surveillance or earlier clinical review; however, further replication is needed in more diverse groups.

Given that a risk factor for PML in persons taking DMF is lymphopenia,^1^ people affected by MS could benefit from the current research in the future, as there is potential for polygenic scores to be used to identify those at highest risk of an adverse drug reaction. They could be offered a different treatment, alternative dosing or more frequent monitoring, depending on their polygenic risk for lymphocyte counts. Inherited potential for low lymphocyte counts in people with MS is likely also relevant to other therapies associated with lymphopenia, such as ocrelizumab,^22^ and may represent an area of future investigation.

Genetic variation and its association with adverse drug reactions are increasingly recognized and used for risk stratification, with upwards of 90% of people reported to have at least one genotype associated with an actionable pharmacogenetic test.^23^ Here, we identified a genome-wide significant locus on chromosome 9 associated with the minimum lymphocyte count during DMF treatment in the discovery cohort. This variant has not been previously reported in the literature, nor in the GWAS catalogue, which includes over 7000 publications reporting GWAS findings.^24^ Although this finding did not replicate in the Swedish cohort, this could be due to differences between the individuals included in the two cohorts and different requirements for bloodwork monitoring between our study regions and would require replication in an additional cohort before proceeding with potential clinical applications.

A previous study investigated the genetic determinants of DMF-induced lymphopenia in 1258 people with MS from the DEFINE/CONFIRM phase 3 clinical trials, defining outcomes as sustained grade 3 (absolute lymphocyte counts <0.5 × 10^9^/L) or grade 2 (absolute lymphocyte counts 0.5 to 0.8 × 10^9^/L) lymphopenia persisting for 6 months or more.^10^ No genome-wide significant variants were identified; however, only approximately 1 million variants were tested, compared with the >8 million included in our study. Moreover, the previous study did not investigate lymphocyte count as a continuous measure, which can improve statistical power, nor did it assess the polygenicity of any traits. We assessed the association of the most significant genetic variant from that previous study with our lymphopenia outcomes, and although the effects were largely in the same direction in our discovery cohort, none reached statistical significance.

Our study had strengths and limitations. Strengths included a sufficiently powered discovery cohort to identify a genetic marker with a clinically significant odds ratio for the primary comparison of DMF-grade 2 lymphopenia. Significantly larger effect sizes have been reported for certain pharmacogenetic phenotypes (i.e. adverse events), compared with studies of other dichotomous complex disease traits.^25^ We included multiple indices of common genetic variation, such as individual genetic variation and cumulative genetic burden measures. We also used existing GWAS evidence from an exceptionally large cohort (i.e. UK Biobank) for the polygenic score analyses and applied them to a smaller, clinically phenotyped cohort of people with MS treated with DMF. Our study had limitations, which included only using participants of European genetic ancestry. We were not powered to identify any genome-wide significant common genetic variation in non-European groups, such as African or Asian populations, as they were each <20 individuals and, therefore, excluded from the analyses. Discovery and replication in non-European genetic ancestry groups would be required in the future to establish generalizability. In our analyses, we considered baseline lymphocyte counts but did not consider other factors potentially associated with lymphopenia in people with MS taking DMF, which include BMI^26,27^ and smoking.^26^ Both smoking and BMI have been associated with altered lymphocyte dynamics, and their inconsistent availability across cohorts represents a limitation. However, because both cases and controls were DMF-treated individuals with MS, confounding by these factors would require them to be differentially distributed between groups to bias our findings, and the use of DMF-tolerant, rather than population controls, provides some inherent protection against this. Our primary aim was to identify novel genetic factors associated with this adverse reaction, rather than to build a comprehensive prediction model, and future studies with more complete phenotypic data should examine whether BMI, smoking and genetic liability act independently or interactively in determining lymphopenia risk during DMF treatment.

The lack of significant MAGMA gene-based or pathway enrichment results should be interpreted considering the small GWAS sample size. These analyses are, therefore, underpowered and should be considered exploratory. The top-ranked genes and nominal pathway signals may be hypothesis-generating, but larger cohorts are required before drawing firm biological conclusions.

In conclusion, we found that common genetic variation, indexed by a cumulative genetic liability measure, was associated with low lymphocyte counts and lymphopenia in people with MS of European genetic ancestry treated with DMF. Ultimately, the findings from this study could improve the treatment of MS by identifying who is most likely to have a potentially serious reduction in lymphocytes before they occur and thus enhance the health of people with MS. The implications are also beyond those of MS, as we found polygenicity for a biological trait (lymphocyte levels) was associated with sensitizing an individual to drug-related disruption of that same trait.

## Supplemental Material

sj-docx-1-msj-10.1177_13524585261471335 – Supplemental material for Lymphocyte count polygenicity underlies dimethyl fumarate–associated lymphopenia in multiple sclerosis Supplemental material, sj-docx-1-msj-10.1177_13524585261471335 for Lymphocyte count polygenicity underlies dimethyl fumarate–associated lymphopenia in multiple sclerosis by Kaarina Kowalec, Ali Manouchehrinia, Elaine Kingwell, Robert Carruthers, Ruth Ann Marrie, Charity Evans, Dalia Rotstein, Annie J Kuan, Wenzhuo Luo, Daniel Luo, Brianne Desrochers, Vilija G Jokubaitis, Jeannette Lechner-Scott, Vicki E Maltby, Kira Groen, Galen EB Wright, Britt I Drögemöller, Sasha Bernatsky, Michael C Levin, Katherine Knox, Abdullah Al Maruf, Michael Zhong, Lars Alfredsson, Jan Hillert, Ingrid Kockum, Tomas Olsson, Pernilla Stridh, Klementy Shchetynsky, Kristy Dever, Julie MacIsaac, Michael S Kobor and Helen Tremlett in Multiple Sclerosis Journal

## Data Availability

Individual participant data collected for the study cannot be shared, but access may be granted via authorization from the study investigators with the appropriate ethical approvals and data-sharing agreements. Summary-level GWAS statistics can be accessed via requests to Dr K.K.
