# Supplementary material for: Lymphocyte count polygenicity underlies dimethyl fumarate–associated lymphopenia in multiple sclerosis
Source: Mult Scler. 2026 Aug 17;32(12):1292–301. doi: 10.1177/13524585261471335 (PMC13672884; doi:10.1177/13524585261471335)
Supplement: Supplementary file 1 [file sj-docx-1-msj-10.1177_13524585261471335.docx]

## **Online-only**

## **Standard Protocol Approvals and Patient Consent**

All participants provided informed consent. The study was approved by the respective site Health Research Ethics Boards (University of British Columbia, University of Saskatchewan, University of Manitoba, University of Toronto, Hunter New England Local Health District Human Research Ethics Committee, Melbourne Health Human Research Ethics Committee) and Health Authorities (Vancouver Coastal Health, Shared Health [Manitoba], Unity Health Toronto). In Sweden, the study was originally approved by the Swedish Ethical Review Authority.

### ***Design***

## In the current study, we improve the original protocol to reflect evolutions to current best practices for GWAS, including polygenic score analyses, applying a stricter genome-wide significance threshold (p<5e-08 instead of p<1e-05), and using the latest genetic analyses software.

### ***Discovery cohort: Canada and Australia***

Participants were recruited from three tertiary care clinics for MS in Canada: The Djavad Mowafaghian Centre for Brain Health (Vancouver, British Columbia), Health Sciences Centre Winnipeg (Winnipeg, Manitoba), and St. Michael's Hospital (Toronto, Ontario) between 2017-2022. Additionally, potential participants were identified and recruited from the Saskatchewan MS Drugs Research Program, which supports study of the utilization of MS disease-modifying therapies by those with MS in Saskatchewan. All individuals provided a saliva sample for DNA extraction (saliva collection kits: DNA Genotek, Ottawa, Canada). Phenotype data were collected using a standardized report form and included drug exposure (DMF exposure dates), laboratory test results (pre-DMF and during DMF laboratory test results, along with the corresponding normal limits for absolute lymphocyte counts), and demographic information (sex, age) from medical records. For Australia, we withdrew DNA from existing studies that had collected samples from people with MS from two tertiary MS outpatient centres: The John Hunter Hospital (Newcastle, New South Wales) and the Alfred Hospital (Melbourne, Victoria). Newcastle DNA samples were sourced from the biospecimen bank at the John Hunter Hospital. Alfred Hospital samples have been previously described, with DNA derived from blood.^1^ Like the Canada cohort, all individuals were exposed to DMF, with clinical, laboratory test results, and demographic data retrieved from medical records. Samples from Canada and Australia participants were pooled together when generating genetic data.

***Replication cohort: Sweden***

Participants exposed to DMF for MS in Sweden were identified from the Immunomodulation and MS Epidemiology (IMSE) study. IMSE is a nationwide post-marketing cohort established within the Swedish MS registry to monitor the long-term safety and effectiveness of new disease-modifying therapies. Although participation in the IMSE study is voluntary, coverage is highly comprehensive, with most patients initiating modern MS therapies included.^13^ Like the Canadian and Australian participants, all individuals in Sweden were exposed to DMF, with clinical, laboratory test results, and demographic data retrieved from medical records, IMSE database, or the Swedish MS Registry (SMSReg).^14^

***Genotyping, quality control, and imputation***

For the discovery cohort, DNA was either extracted from saliva (Canada) or was already available (Australia) and then genotyped using the Illumina Global Screening Array (v3), including 699,424 markers. Quality control of the genotypes was conducted using a well-known genetics consortium pipeline, RICOPILI (Rapid Imputation for COnsortias PIpeLIne).^2^ Briefly, we excluded the following participant samples: genotype missingness >0.02 (after first filtering variants with call rate <0.95), genetic sex not matching phenotypic data, autosomal heterozygosity >0.2, or in the case of related individuals, one sample in each related pair was excluded. We excluded one individual from each related pair in the discovery cohort based on pairwise genetic relatedness estimates (PI_HAT>[0.1/0.2]), preferentially retaining DMF lymphopenia cases. We removed variants with a call rate <0.99, difference in missingness between cases and controls >0.005, minor allele frequency <0.01, ambiguous variants, or deviating from Hardy-Weinberg equilibrium in cases or controls (P<10^-6^) leaving 522,343 variants for imputation. We performed whole-genome imputation to include an exhaustive analysis of common genetic variation. For imputation, we used two reference panels: 1) Trans-Omics for Precision Medicine (TOPMed, version r3)^3^ for the GWAS and polygenic score analyses, and 2) 1000 Genomes Project^4^ for genetic ancestry determination. Following imputation, TOPMed imputation provided 307,616,075 variants, and we removed any variants with poor imputation (imputed information [INFO] score ≤0.7) or that were rare (minor allele frequency <0.01), leaving 9,207,020 variants for analysis. Similar metrics and steps were taken for cleaning the genotyping data from the replication cohort in Sweden, except that they were genotyped on two different genotyping arrays (Illumina Global Screening Array v1 or Illumina Human OmniExpress array) and were analyzed separately and then meta-analyzed using PLINK1.9^5^ with an inverse-variance weighted fixed-effects model. Genotype imputation for the Swedish participants was performed using the Haplotype Reference Consortium (version r1.1) as described previously.^6^ To minimize confounding, we restricted the analyses to those of European genetic ancestry.^7^ Genetic ancestry was confirmed using principal components analysis,^8^ with participants excluded based on site-specific cut-offs that were >3 (Canada/Australia) or >6 (Sweden; more homogeneous sample so more liberal cut-off used^9^) standard deviations away from the European mean principal component 1 or 2 using 1000 Genomes Project (European)^4^ as the reference. Non-European participants were then excluded, and the principal components were re-generated to be used as covariates in the analyses to adjust for any residual population stratification.

These GWAS training data were harmonized to the genotype “test data” (that is, the individual level genotypes from our current study) by aligning to the same effect-increasing allele. Bayesian polygenic score methods such as SBayesR are preferred over earlier methods, such as p-value thresholding and clumping, as they more effectively integrate genome-wide information and account for linkage disequilibrium and effect size shrinkage.^10^ Polygenic scores show a marked inflection at the upper ends of the distribution, with those in highest quantiles often compared to those in the lowest quantiles;^11^ thus, for ease of interpretation, we have included the polygenic score as a continuous measure and as a quantile measure (top 20% lymphocyte count genetic liability vs. remaining 80%).^12^

**Gene-based and pathway enrichment analyses**

To complement the single-variant GWAS results, we performed gene-based association testing using MAGMA. SNP-level GWAS summary statistics were mapped to genes using the corresponding genome-build gene annotation and a 1000 Genomes reference panel for linkage disequilibrium estimation. Gene-based tests were conducted across 18,051 genes. Gene-level significance was assessed using both Bonferroni correction and Benjamini-Hochberg false discovery rate correction. We then performed competitive gene-set enrichment analyses using MAGMA on the gene-level results. Gene sets were obtained from MSigDB, including Hallmark, Reactome, and Gene Ontology collections. Enrichment results were evaluated within each collection and across all tested gene sets using multiple-testing correction. These analyses were considered exploratory and were used to assess whether association signals showed evidence of convergence on shared biological pathways rather than relying only on individual SNP-level loci.

**Statistical Analyses**

We reported the descriptives of participants by discovery and replication cohorts.

For the GWAS, an *a priori* sample size calculation determined that we were 80% powered to identify a genetic marker with a clinically significant per-allele odds ratio of >5 ^13^ for the primary comparison (Grade 2 lymphopenia) with >68 cases (assuming minor allele frequency=10%, p<5e-08). Power increases as variants become more common; however, across minor allele frequencies of 15–30%, the odds ratio required for 80% power remains greater than approximately 6. Accordingly, the study is primarily powered to detect large effect sizes and has limited power to detect modest genetic effects typical of complex traits. Power is further reduced for the Grade 3 lymphopenia analyses due to the smaller number of events.

Any variants with an association reaching the standard genome-wide significance threshold (p<5x10^-8^) in the discovery cohort were summarized as the number of linkage disequilibrium-independent (r^2^<0.1 in 3Mb windows) index variants. Loci are then defined as the linkage disequilibrium-independent region within a 50-kb window (i.e., collapsing to one locus). Any genome-wide significant variants were then harmonized for assessment (ensuring both cohorts defined effects relative to the same allele) in the replication cohort, with any variants reaching a significance threshold of p≤0.05 subject to annotation to understand the functional role and significance of the variants. Replication association analyses in the Swedish cohort were performed for log-transformed minimum lymphocyte count using linear regression, adjusted for sex, age, and genetic principal components. Phenotype-matched replication GWAS for the binary grade-specific and prolonged lymphopenia outcomes was not performed. If there were no genome-wide significant variants associated with the respective outcome, we reported the first ten variants, by lowest p-value, to be used for future research studies. Last, we assessed the effect of the four categorical study outcomes in the discovery cohort of a variant previously associated with DMF-associated lymphopenia which did not reach the standard genome-wide significance threshold (rs12249496, p=9.9x10^-7^) in that study.^14^

For the GWAS, we tested the association of each genetic marker with case/control status or minimum lymphocyte count using regression, with adjustment for the first five genetic ancestry principal components (for residual confounding by population stratification). For the categorical outcomes (grade 2, grade 3, prolonged grade 2, and prolonged grade 3), we used binary logistic regression in PLINK2 with additive genotype coding (0, 1, or 2 copies of the effect allele). We also examined the minimum lymphocyte count during DMF treatment as a continuous outcome using linear regression in PLINK2.^15^ We also examined the minimum lymphocyte count during DMF treatment as a continuous outcome. For this analysis, we used the log-transformed minimum lymphocyte count as the phenotype in the PLINK2 linear regression analyses. We reported the odds ratio (and 95% confidence intervals) for categorical, and beta estimates (and standard errors) for continuous outcomes.

PLINK1.9, PLINK2 and *R* for Statistical Computing (v4.4.3) and *R*-Studio (v2024.12.1+563) were used for analyses with the following packages: *tidyverse*, *data.table, icenReg*.

Online references

1.	Jokubaitis VG, Campagna MP, Ibrahim O, et al. Not all roads lead to the immune system: the genetic basis of multiple sclerosis severity. *Brain*. Published online November 30, 2022. doi:10.1093/BRAIN/AWAC449

2.	Lam M, Awasthi S, Watson HJ, et al. RICOPILI: Rapid Imputation for COnsortias PIpeLIne. Schwartz R, ed. *Bioinformatics*. Published online August 8, 2019. doi:10.1093/bioinformatics/btz633

3.	Das S, Forer L, Schönherr S, et al. Next-generation genotype imputation service and methods. *Nat Genet*. 2016;48(10):1284-1287. doi:10.1038/ng.3656

4.	1000 Genomes Project Consortium, Auton A, Brooks LD, et al. A global reference for human genetic variation. *Nature*. 2015;526(7571):68-74. doi:10.1038/nature15393

5.	Purcell S, Neale B, Todd-Brown K, et al. PLINK: a tool set for whole-genome association and population-based linkage analyses. *Am J Hum Genet*. 2007;81(3):559-575. doi:10.1086/519795

6.	International Multiple Sclerosis Genetics Consortium, MultipleMS Consortium. Locus for severity implicates CNS resilience in progression of multiple sclerosis. *Nature*. Published online June 28, 2023. doi:10.1038/s41586-023-06250-x

7.	Martin AR, Kanai M, Kamatani Y, Okada Y, Neale BM, Daly MJ. Clinical use of current polygenic risk scores may exacerbate health disparities. *Nat Genet*. 2019;51(4):584-591. doi:10.1038/s41588-019-0379-x

8.	Price AL, Patterson NJ, Plenge RM, Weinblatt ME, Shadick NA, Reich D. Principal components analysis corrects for stratification in genome-wide association studies. *Nat Genet*. 2006;38(8):904-909. doi:10.1038/ng1847

9.	Holmen C, Piehl F, Hillert J, et al. A Swedish national post-marketing surveillance study of natalizumab treatment in multiple sclerosis. *Mult Scler*. 2011;17(6):708-719. doi:10.1177/1352458510394701

10.	Lloyd-Jones LR, Zeng J, Sidorenko J, et al. Improved polygenic prediction by Bayesian multiple regression on summary statistics. *Nat Commun*. 2019;10(1):5086. doi:10.1038/s41467-019-12653-0

11.	Choi SW, Mak TSH, O’Reilly PF. Tutorial: a guide to performing polygenic risk score analyses. *Nat Protoc*. 2020;15(9):2759-2772. doi:10.1038/s41596-020-0353-1

12.	Cupido AJ, Tromp TR, Hovingh GK. The clinical applicability of polygenic risk scores for LDL-cholesterol: considerations, current evidence and future perspectives. *Curr Opin Lipidol*. 2021;32(2):112-116. doi:10.1097/MOL.0000000000000741

13.	Maranville JC, Cox NJ. Pharmacogenomic variants have larger effect sizes than genetic variants associated with other dichotomous complex traits. *Pharmacogenomics J*. 2015;16(4):388-392. doi:10.1038/tpj.2015.47

14.	Sangurdekar D, Sun C, McLaughlin H, et al. Genetic Study of Severe Prolonged Lymphopenia in Multiple Sclerosis Patients Treated With Dimethyl Fumarate. *Front Genet*. 2019;10:1039. doi:10.3389/fgene.2019.01039

**Figure S1:** Principal components (PC) analysis of participants for genetic ancestry determination using PC1 and PC2 in the discovery cohort of study participants with multiple sclerosis and exposed to dimethyl fumarate along with 1000 Genomes Project reference data.

**Figure S2:** Quantile-quantile (Q-Q) plot of the genome-wide association results for the lowest absolute lymphocyte counts during dimethyl fumarate treatment in people with multiple sclerosis in the discovery cohort.

**Figure S3:** Regional association plot for lowest absolute lymphocyte counts during dimethyl fumarate treatment in people with multiple sclerosis in the discovery cohort. X-axis: chromosome position (kb), Primary y-axis: significance of association (log10(p-value), Secondary y-axis: recombination rate (%). The blue dashed line shows the genome-wide significance level (p=5x10^-8^). The purple diamond indicates the lead variant (rs17087205). Colour scheme: Legend for linkage disequilibrium r^2^ is given in the left corner, with grey indicating no LD information available. Genes in the lower part of the plot are annotated based on University of California Santa Cruz (UCSC) Genome Browser with navy vertical lines designating the exons. All coordinates are in genome build GRCh38. The blue vertical lines denote regional recombination rates derived from HapMap. Figure produced using *locuszoomr* R-package.

**Figure S4:** Quantile-quantile (Q-Q) plot of genome-wide association results for Grade two lymphopenia during dimethyl fumarate treatment in people with multiple sclerosis in the discovery cohort.

**Figure S5:** Regional association plot for lowest absolute lymphocyte counts during dimethyl fumarate treatment in people with multiple sclerosis in the Swedish cohort. X-axis: chromosome position (kb), Primary y-axis: significance of association (log10(p-value), Secondary y-axis: recombination rate (%). The purple diamond indicates the lead variant in the Discovery analyses (rs17087205). All coordinates are in genome build GRCh37. Colour scheme: Legend for linkage disequilibrium r^2^ is given in the left corner, with grey indicating no LD information available. Genes in the lower part of the plot are annotated based on University of California Santa Cruz (UCSC) Genome Browser with navy vertical lines designating the exons. The blue vertical lines denote regional recombination rates derived from HapMap. Figure produced using *locuszoomr* R-package.

### **Figure S6:** Manhattan plot depicting the genetic association results for Grade two lymphopenia during dimethyl fumarate treatment in people with multiple sclerosis in the Discovery cohort. X-axis: Chromosome number. Y-axis: -log10(p-value). The black dashed line indicates the genome-wide significance threshold (p<5x10^-8^).

### **Table S1:** Cox regression results investigating the association between baseline lymphocyte counts and polygenic scores with the risk of developing grades of lymphopenia during dimethyl fumarate treatment in people with MS.

| **Cohort** | **Outcome** | **Term** | **Hazard Ratio (95%CI)** | **P-value** |
| --- | --- | --- | --- | --- |
| Model 1-Discovery | Grade 2 lymphopenia | Baseline lymphocyte count | 0.54 (0.29-1.01) | 0.053 |
| Model 2-Discovery |  | Baseline lymphocyte count | 0.60 (0.31-1.14) | 0.119 |
|  |  | Polygenic score | 0.48 (0.27-0.87) | 0.016 |
| Model 1-Replication |  | Baseline lymphocyte count | 0.45 (0.20-1.03) | 0.058 |
| Model 2-Replication |  | Baseline lymphocyte count | 0.61 (0.24-1.55) | 0.299 |
|  |  | Polygenic score | 0.37 (0.10-1.31) | 0.124 |
| Model 3-Discovery | Grade 3 lymphopenia | Baseline lymphocyte count | 0.49 (0.26-0.92) | 0.027 |
| Model 4-Discovery | Grade 3 lymphopenia | Baseline lymphocyte count | 0.53 (0.29-0.98) | 0.045 |
|  |  | Polygenic score | 0.58 (0.24-1.39) | 0.221 |

Each model includes age, sex and the first five genetic ancestry principal components, in addition to those listed in the Term column. Grade 3 lymphopenia model in Sweden did not converge with only 2 events.

**Table S2**: Top independent discovery lead variants for log-transformed minimum lymphocyte count during dimethyl fumarate treatment and corresponding Swedish results. CADD PHRED: Combined Annotation Dependent Depletion PHRED-scaled score; higher scores indicate greater predicted functional impact.

| **CHR** | **Position** | **Effect allele** | **Effect allele frequency** | **Beta coefficient** | **Standard error** | **P-value** | **Swedish Beta coefficient** | **Swedish Standard error** | **Swedish P-value** | **Primary consequence** | **Nearest gene** | **CADD PHRED** |
| --- | --- | --- | --- | --- | --- | --- | --- | --- | --- | --- | --- | --- |
| 9 | 84417739 | G | 0.054852 | -0.62643 | 0.109731 | 3.52E-08 | -0.05159 | 0.09835 | 0.599873 | intron | *LOC124902192* | 1.295 |
| 8 | 19902655 | C | 0.014768 | -1.10035 | 0.202449 | 1.4E-07 | N/A | N/A | N/A | intron | *LPL* | 0.354 |
| 17 | 6761653 | G | 0.037975 | -0.64924 | 0.125274 | 4.82E-07 | 0.110509 | 0.154514 | 0.474481 | promoter | *XAF1* | 0.081 |
| 2 | 166353009 | GTT | 0.139241 | -0.35957 | 0.070514 | 7.16E-07 | N/A | N/A | N/A | intron | *SCN9A* | 1.007 |
| 7 | 153112920 | G | 0.025317 | -0.80086 | 0.159547 | 1.04E-06 | N/A | N/A | N/A | intergenic | *LINC01287* | 8.953 |
| 22 | 44612968 | T | 0.025317 | -0.80011 | 0.162094 | 1.54E-06 | N/A | N/A | N/A | intron | *LINC00229* | 5.004 |
| 14 | 90975930 | C | 0.014768 | -1.03366 | 0.210588 | 1.75E-06 | -0.23826 | 0.165466 | 0.149882 | intron | *RPS6KA5* | 0.024 |
| 12 | 78476854 | T | 0.14346 | -0.35159 | 0.071679 | 1.78E-06 | -0.0323 | 0.070807 | 0.648255 | intron | *LINC02424* | 3.939 |
| 5 | 79852499 | C | 0.031646 | -0.66674 | 0.136347 | 1.9E-06 | -0.12327 | 0.158864 | 0.437783 | intergenic | *LOC102724557* | 0.216 |
| 1 | 117797335 | A | 0.014768 | -0.9858 | 0.205435 | 2.89E-06 | N/A | N/A | N/A | intergenic | *GDAP2* | 2.767 |

### **Table S3:** Top genetic associations for Grade 2 lymphopenia during dimethyl fumarate treatment in people with multiple sclerosis in the discovery cohort. All coordinates are in genome build GRCh38.

| **CHR** | **Position** | **Effect**  **allele** | **Effect allele**  **frequency** | **Odds**  **ratio** | **Standard**  **error** | **P-value** | **Primary**  **consequence** | **Nearest**  **gene** | **CADD**  **PHRED** |
| --- | --- | --- | --- | --- | --- | --- | --- | --- | --- |
| 3 | 53993027 | A | 0.451087 | 0.327274 | 0.255217 | 1.20595E-05 | intron | *LOC105377095* | 2.362 |
| 13 | 42201919 | G | 0.274457 | 3.70334 | 0.300924 | 1.35687E-05 | intron | *DGKH* | 2.954 |
| 5 | 79582556 | C | 0.3125 | 0.263891 | 0.312827 | 2.05669E-05 | intron | *LOC105379047* | 0.597 |
| 5 | 122695628 | C | 0.179348 | 4.08973 | 0.331266 | 2.12052E-05 | intron | *LINC02201* | 1.318 |
| 18 | 67624021 | T | 0.0869565 | 8.3139 | 0.498708 | 2.16817E-05 | intron | *DSEL-AS1* | 0.555 |
| 8 | 73482676 | G | 0.326087 | 3.27393 | 0.281022 | 2.43998E-05 | intergenic | *STAU2* | 0.622 |
| 5 | 3770486 | A | 0.171196 | 4.0979 | 0.334734 | 2.51206E-05 | intergenic | *LOC105374625* | 0.011 |
| 7 | 32037403 | C | 0.274457 | 3.33584 | 0.286423 | 2.59807E-05 | intron | *PDE1C* | 3.741 |
| 4 | 184411938 | A | 0.252717 | 3.6198 | 0.305885 | 2.60438E-05 | intron | *IRF2* | 1.836 |
| 13 | 28595299 | AT | 0.459239 | 2.81264 | 0.247232 | 2.87924E-05 | intron | *LOC105370135* | 0.185 |

### **Table S4:** Top genetic associations for Grade 3 lymphopenia during dimethyl fumarate treatment in people with multiple sclerosis in the discovery cohort. All coordinates are in genome build GRCh38.

| **CHR** | **Position** | **Effect**  **allele** | **Effect allele**  **frequency** | **Odds**  **ratio** | **Standard**  **error** | **P-value** | **Primary**  **consequence** | **Nearest**  **gene** | **CADD**  **PHRED** |
| --- | --- | --- | --- | --- | --- | --- | --- | --- | --- |
| 11 | 93556766 | AT | 0.195205 | 6.06308 | 0.417157 | 1.55861E-05 | promoter | *SMCO4* | 4.336 |
| 18 | 27827882 | G | 0.34589 | 4.41728 | 0.344464 | 1.61371E-05 | intron | *LOC105372042* | 1.637 |
| 5 | 80514476 | AAAT | 0.363014 | 4.74967 | 0.372007 | 2.81052E-05 | intron | *FAM151B* | NA |
| 7 | 129569069 | A | 0.452055 | 0.20909 | 0.380575 | 3.91953E-05 | intergenic | *NRF1* | 1.56 |
| 4 | 38720114 | G | 0.455479 | 5.14643 | 0.399732 | 4.15837E-05 | promoter | *KLF3* | 0.143 |
| 5 | 3770486 | A | 0.14726 | 5.57085 | 0.421631 | 4.62954E-05 | intergenic | *LOC105374625* | 0.011 |
| 7 | 87564281 | A | 0.267123 | 4.08805 | 0.347591 | 5.10139E-05 | intergenic | *ABCB1* | 4.034 |
| 1 | 85489578 | A | 0.140411 | 7.26139 | 0.48967 | 5.14833E-05 | intron | *DDAH1* | 3.362 |
| 14 | 20027032 | T | 0.465753 | 4.57131 | 0.375624 | 5.20851E-05 | intergenic | *OR4N2* | 0.62 |
| 22 | 26292038 | T | 0.421233 | 0.193266 | 0.406265 | 5.21336E-05 | promoter | *SEZ6L* | 0.452 |

### **Table S5:** Top genetic associations for Grade 2 prolonged lymphopenia during dimethyl fumarate treatment in people with multiple sclerosis in the discovery cohort. All coordinates are in genome build GRCh38.

| **CHR** | **Position** | **Effect**  **allele** | **Effect allele**  **frequency** | **Effect**  **estimate** | **Standard**  **error** | **P-value** | **Primary**  **consequence** | **Nearest**  **gene** | **CADD**  **PHRED** |
| --- | --- | --- | --- | --- | --- | --- | --- | --- | --- |
| 13 | 75871264 | T | 0.124204 | 11.1636 | 0.520461 | 3.55877E-06 | intron | *LMO7DN* | NA |
| 3 | 59380392 | G | 0.117834 | 8.2013 | 0.459252 | 4.60542E-06 | intron | *CFAP20DC-DT* | 0.489 |
| 12 | 78476854 | T | 0.140127 | 7.16585 | 0.44571 | 9.94336E-06 | intron | *LINC02424* | 3.939 |
| 4 | 152113018 | G | 0.0764331 | 14.615 | 0.612496 | 1.19287E-05 | intergenic | *LINC02273* | 1.924 |
| 7 | 38497248 | T | 0.442675 | 3.80754 | 0.307739 | 1.39569E-05 | intergenic | *AMPH* | 0.94 |
| 9 | 84443287 | T | 0.0509554 | 63.3805 | 0.955235 | 1.40168E-05 | intron | *LOC124902192* | 0.714 |
| 13 | 79386591 | T | 0.484076 | 0.259958 | 0.32015 | 2.57482E-05 | intron | *RBM26* | NA |
| 7 | 28314886 | G | 0.207006 | 4.53263 | 0.361781 | 2.94862E-05 | intron | *CREB5* | 1.039 |
| 9 | 115816554 | T | 0.159236 | 5.26687 | 0.398489 | 3.05482E-05 | intron | *LOC105376234* | 2.705 |
| 3 | 126186309 | C | 0.468153 | 0.252118 | 0.332083 | 3.33727E-05 | intron | *ALDH1L1* | 8.64 |

### **Table S6:** Top genetic associations for Grade 3 prolonged lymphopenia during dimethyl fumarate treatment in people with multiple sclerosis in the discovery cohort. All coordinates are in genome build GRCh38. CADD PHRED: Combined Annotation Dependent Depletion PHRED-scaled score; higher scores indicate greater predicted functional impact

| **CHR** | **Position** | **Effect**  **allele** | **Effect allele**  **frequency** | **Effect**  **estimate** | **Standard**  **error** | **P-value** | **Primary**  **consequence** | **Nearest**  **gene** | **CADD**  **PHRED** |
| --- | --- | --- | --- | --- | --- | --- | --- | --- | --- |
| 10 | 53134589 | A | 0.0731707 | 28.9421 | 0.824572 | 4.47906E-05 | intergenic | *LINC02672* | 0.401 |
| 11 | 113718584 | T | 0.0569106 | 37.3242 | 0.901872 | 5.98313E-05 | intergenic | *TMPRSS5* | 0.161 |
| 5 | 167557358 | C | 0.0691057 | 50.5634 | 0.995318 | 8.09121E-05 | intergenic | *TENM2* | 1.9 |
| 8 | 82706587 | TACAC | 0.0609756 | 36.4784 | 0.925089 | 0.000101085 | intergenic | *LOC105375931* |  |
| 7 | 132900767 | A | 0.0365854 | 41.7202 | 0.970785 | 0.000121407 | intron | *CHCHD3* | 0.459 |
| 10 | 113367695 | CA | 0.0528455 | 30.4966 | 0.892277 | 0.000128031 | intergenic | *LOC107984270* | 0.312 |
| 7 | 101508653 | G | 0.0934959 | 21.8443 | 0.80575 | 0.000129494 | intron | *COL26A1* | 1.451 |
| 8 | 93030881 | A | 0.0731707 | 19.6087 | 0.778704 | 0.000132536 | intergenic | *MIR8084* | 1.022 |
| 3 | 150087896 | C | 0.0406504 | 34.3062 | 0.926307 | 0.000135314 | intron | *LOC105374313* | 0.87 |
| 5 | 63334629 | C | 0.0365854 | 30.5753 | 0.897077 | 0.000137515 | intron | *HTR1A* | 3.787 |

**Table S7:** Assessment of previously reported genetic associations [1] in the context of lymphopenia findings from the current study [2] following dimethyl fumarate treatment in people with multiple sclerosis.

| **[1] Sangurdekar et al. (2019)**^14^ | | **[2] Current study** | | | |
| --- | --- | --- | --- | --- | --- |
| **Allele 1 Frequency** | **Odds ratio (95%CI), P** | **Comparison** | **Allele 1 Frequency** | **Odds ratio (95%CI)** | **P** |
| Not reported | 2.2 (1.6-3.0), 9.9e-07 | Moderate/Grade 2 Lymphopenia vs. DMF-tolerant controls | 0.147 | 1.11 (0.61-2.02) | 0.729 |
|  |  | Severe/Grade 3 Lymphopenia vs. DMF-tolerant controls | 0.151 | 1.31 (0.62-2.77) | 0.477 |
|  |  | Moderate/Grade 2 prolonged Lymphopenia vs. DMF-tolerant controls | 0.142 | 1.01 (0.27-3.72) | 0.986 |
|  |  | Severe/Grade 3 prolonged Lymphopenia vs. DMF-tolerant controls | 0.134 | 0.77 (0.36-1.66) | 0.512 |

Details of genetic variant previously associated with moderate or severe prolonged lymphopenia: rs12249496, Coordinates: chr10: 130673804 (GRCh37), chr10: 128875540 (GRCh38). DMF: dimethyl fumarate.

**Table S8:** Exploratory gene-based association results from MAGMA analysis for dimethyl-fumarate associated minimum lymphocyte counts in MS.

| **Gene** | **Gene name** | **Entrez ID** | **Chromo-some** | **Start position** | **Stop position** | **Number of SNPs** | **Z-Statistic** | **P-value** | **Bonferroni False discovery rate** |
| --- | --- | --- | --- | --- | --- | --- | --- | --- | --- |
| *PRKAG3* | Protein kinase AMP-activated non-catalytic subunit gamma 3 | 53632 | 2 | 219687083 | 219696526 | 11 | 4.0225 | 2.88e-05 | 0.339 |
| *LPL* | Lipoprotein lipase | 4023 | 8 | 19796582 | 19824770 | 132 | 3.9594 | 3.76e-05 | 0.339 |
| *SCN9A* | Sodium voltage-gated channel alpha subunit 9 | 6335 | 2 | 167051695 | 167232497 | 702 | 3.6834 | 1.15e-04 | 0.569 |
| *LILRB2* | Leukocyte immunoglobulin like receptor B2 | 10288 | 19 | 54777675 | 54785033 | 22 | 3.6598 | 1.26e-04 | 0.569 |
| *SLC28A3* | Solute carrier family 28 member 3 | 64078 | 9 | 86890765 | 86983413 | 458 | 3.4961 | 2.36e-04 | 0.739 |
|  |  |  |  |  |  |  |  |  |  |
